# Supplementary material for: Salivary and plasmatic oxytocin are not reliable trait markers of the physiology of the oxytocin system in humans
Source: eLife. 2020 Dec 11;9:e62456. doi: 10.7554/eLife.62456 (PMC7732341; doi:10.7554/eLife.62456)
Supplement: Supplementary file 1. — ICC – Intraclass correlation coefficient; CV – coefficient of variation; CI – confidence interval; SD – Standard Deviation; *H0: ICC is not significantly different from 0; statistical significance was set to p<0.05 (two-tailed). N represents the actual size of the sample used to calculate the ICCs and the CVs. Table 2 – Absolute and relative between-visits reliability of oxytocin measurements in plasma and saliva controlling for time-interval between visits (dataset A). ICC – Intraclass correlation coefficient; CV – coefficient of variation; CI – confidence interval; SD – Standard Deviation; *H0: ICC is not significantly different from 0; statistical significance was set to p<0.05 (two-tailed). N represents the actual size of the sample used to calculate the ICCs and the CVs. Table 3 – Absolute and relative within-visit reliability of oxytocin measurements in plasma and saliva in the placebo visit of dataset A. ICC – Intraclass correlation coefficient; CV – coefficient of variation; CI – confidence interval; SD – Standard Deviation; *H0: ICC is not significantly different from 0; statistical significance was set to p<0.05 (two-tailed). N represents the actual size of the sample used to calculate the ICCs and the CVs. [file elife-62456-supp1.docx]

# Supplementary file 1

**Table 1.**

|  |  |  | N | ICC | | | | | CVMean (SD) | Pearson rr (p-value) |
| --- | --- | --- | --- | --- | --- | --- | --- | --- | --- | --- |
|  |  |  |  |  | 95% CI | | F test* | p-value |  |  |
|  |  |  |  |  | Lower | Upper |  |  |  |  |
| Visit 1 vs 2 | Plasma | Single | 16 | 0.80 | 0.51 | 0.93 | F(15, 15) = 8.45 | <1.00x10^-3^ | 31% (20) | 0.79 (p<0.001) |
|  | Saliva | Single | 13 | 0.11 | -0.51 | 0.62 | F(12, 12) = 1.23 | 0.36 | 39% (38) | 0.11 (0.73) |
| Visit 1 vs 3 | Plasma | Single | 16 | 0.25 | -0.28 | 0.66 | F(15, 15) = 1.64 | 0.18 | 43% (36) | 0.24 (0.36) |
|  | Saliva | Single | 13 | 0.08 | -0.54 | 0.60 | F(12, 12) = 1.15 | 0.41 | 41% (33) | 0.07 (0.82) |
| Visit 1 vs 4 | Plasma | Single | 16 | 0.08 | -0.44 | 0.55 | F(15, 15) = 1.17 | 0.51 | 55% (39) | 0.09 (0.75) |
|  | Saliva | Single | 13 | -0.17 | -0.67 | 0.42 | F(12, 12) = 0.72 | 0.71 | 57% (45) | -0.18 (0.55) |
| Visit 2 vs 3 | Plasma | Single | 16 | 0.09 | -0.43 | 0.56 | F(15, 15) = 1.19 | 0.37 | 51% (38) | 0.09 (0.75) |
|  | Saliva | Single | 13 | 0.82 | 0.52 | 0.94 | F(12, 12) = 9.82 | <1.00x10^-3^ | 31% (31) | 0.83 (p<0.001) |
| Visit 2 vs 4 | Plasma | Single | 16 | -7.00x10^-3^ | -0.50 | 0.48 | F(15, 15) = 0.99 | 0.51 | 57% (38) | -0.01 (0.98) |
|  | Saliva | Single | 13 | 0.35 | -0.21 | 0.74 | F(12, 12) = 2.09 | 0.11 | 47% (39) | 0.38 (0.21) |
| Visit 3 vs 4 | Plasma | Single | 16 | 0.66 | 0.26 | 0.87 | F(15, 15) = 4.69 | 2.00x10^-3^ | 45% (30) | 0.76 (p<0.001) |
|  | Saliva | Single | 13 | 0.30 | -0.24 | 0.71 | F(12, 12) = 1.90 | 0.14 | 47% (41) | 0.36 (0.23) |

# Table 2.

|  | | **N** | **ICC** | | | | | **CV**  **Mean (SD)** |
| --- | --- | --- | --- | --- | --- | --- | --- | --- |
|  |  |  |  | **95% CI** | | **F test*** | **p-value** |  |
|  |  |  |  | **Lower** | **Upper** |  |  |  |
| **Sensitivity analysis** | **Plasma** | 15 | 0.04 | -0.47 | 0.53 | F(14, 14) = 1.08 | 0.45 | 40% (27) |
|  | **Saliva** | 15 | -0.044 | -0.59 | 0.48 | F(14, 14) = 0.92 | 0.56 | 49% (40) |

**Table 3.**

|  | | **N** | **ICC** | | | | | **CV**  **Mean (SD)** |
| --- | --- | --- | --- | --- | --- | --- | --- | --- |
|  |  |  |  | **95% CI** | | **F test*** | **p-value** |  |
|  |  |  |  | **Lower** | **Upper** |  |  |  |
| **Within-visit reliability (placebo)** | **Plasma** | 16 | 0.92 | 0.78 | 0.97 | F(15, 15) = 22.47 | <0.0001 | 22% (23) |
